# Evaluation of Chokeberry/Carboxymethylcellulose Hydrogels with the Addition of Disaccharides: DART-TOF/MS and HPLC-DAD Analysis

**DOI:** 10.3390/ijms24010448

**Published:** 2022-12-27

**Authors:** Ina Ćorković, Aleš Rajchl, Tereza Škorpilová, Anita Pichler, Josip Šimunović, Mirela Kopjar

**Affiliations:** 1 Faculty of Food Technology, Josip Juraj Strossmayer University, F. Kuhača 18, 31000 Osijek, Croatia; 2 Department of Food Preservation, Faculty of Food and Biochemical Technology, University of Chemistry and Technology Prague, Technická 3, Dejvice, 166 28 Prague, Czech Republic; 3 Department of Food, Bioprocessing and Nutrition Sciences, North Carolina State University, Raleigh, NC 27695-7624, USA

**Keywords:** chokeberry, carboxymethylcellulose hydrogels, disaccharides, polyphenols, DART, HPLC

## Abstract

With the growing awareness of the importance of a healthy diet, the need for the development of novel formulations is also on the rise. Chokeberry products are popular among consumers since they are a rich source of polyphenols that are responsible for antioxidant activity and other positive effects on human health. However, other natural food ingredients, such as disaccharides, can affect their stability. The aim of this study was to investigate the influence of disaccharides addition on the polyphenol composition of chokeberry hydrogels. Hydrogels were prepared from chokeberry juice and 2% of carboxymethylcellulose (CMC) with the addition of 30%, 40%, or 50% of disaccharides (sucrose or trehalose). Samples were analyzed using DART-TOF/MS. The method was optimized, and the fingerprints of the mass spectra have been statistically processed using PCA analysis. Prepared samples were evaluated for total polyphenols, monomeric anthocyanins, and antioxidant activity (FRAP, CUPRAC, DPPH, ABTS assays) using spectrophotometric methods. Individual polyphenols were evaluated using HPLC-DAD analysis. Results showed the addition of disaccharides to 2% CMC hydrogels caused a decrease of total polyphenols. These findings confirm proper formulation is important to achieve appropriate retention of polyphenols.

## 1. Introduction

Fresh and unprocessed fruits of black chokeberry (*Aronia melanocarpa*) are infrequently consumed because of their bitter and astringent taste. Therefore, these berries are often processed into juices, jams, fruit teas, sauces, and dietary supplements [1]. Black chokeberries are rich in high-valued bioactive components, such as polyphenols, for which this fruit is the subject of numerous studies [2,3,4]. The most abundant polyphenols in chokeberry juice are anthocyanins (cyanidin-3-galactoside, cyanidin-3-arabinoside), flavonols (rutin), and phenolic acids (neochlorogenic acid) [5]. As a result of high anthocyanin content, chokeberry is commonly used as a dye [6]. A diet rich in polyphenols is associated with numerous positive effects on human health, such as improvement of the lipid balance [7], regulation of hyperglycemia [8], and anticancer [9] and antioxidant activities [10].

One of the most important issues the food industry is currently facing is the development of novel and innovative food products that ensure maximizing the retention of bioactive components with well-known positive effects on human health. Hydrogels are one type of product that can be used for the preservation of polyphenols. These systems are defined as 3D materials composed of polymeric networks that can absorb large amounts of water or other fluids without dissolving and are able to deliver polyphenols [11]. Different biopolymers, such as chitosan, pectin, alginate, and cellulose, are used for the preparation of hydrogels as they are recognized for their low toxicity and a broad range of possible biological properties [12]. Cellulose as the most plentiful biopolymer and its derivatives, such as hydroxyproylcellulose and carboxymethylcellulose, are commonly used as scaffold materials for hydrogels [13]. In the present study, hydrogels for the delivery of chokeberry polyphenols were prepared using carboxymethylcellulose (CMC). This cellulose derivate is familiar for its physiologically inert, odorless, non-caloric, and tasteless properties [14]. CMC hydrogels were used for the formulation of efficient delivery systems of tart cherry polyphenols [15], which resulted in their selection for this study.

It was reported the stability of polyphenols in different type of products and semi-products could be affected by the addition of other food ingredients, including disaccharides, such as sucrose or trehalose. However, works published so far show contradictory results, such as stabilizing and destabilizing effects or no effects of the disaccharides on polyphenols [16,17,18,19,20]. Sucrose is one of the most abundant natural disaccharides with a broad utilization in the food industry. Trehalose is attracting more and more attention as a food additive due to its stability and positive effects on the quality of food products. Additional benefits of this disaccharide cannot be neglected. It digests slowly resulting in a lower glycemic index, with a lower insulin release in comparison to sucrose [21,22]. In comparison to sucrose, it has a lower cariogenic effect and does not possess a laxative effect which was related to other low-cariogenic bulk sweeteners [23]. These properties make trehalose a valuable additive to food products.

Sucrose and trehalose were added to the matrix to investigate their effect on total polyphenols, monomeric anthocyanins, individual polyphenols, and antioxidant activity in prepared hydrogels. Several analytical methods are applied for the detection of polyphenols from chokeberry, such as high-performance liquid chromatography-diode array detection (HPLC-DAD) [24], high-performance liquid chromatography/mass spectrometry (HPLC-MS/MS) [25], high-performance liquid chromatography coupled with diode array detection and electrospray ionization-mass spectrometry (HPLC/DAD/ESI-MS) [26], ultra-performance liquid chromatography, and photodiode detector-quadrupole/time-of-flight mass spectrometry (UPLC-PDA-MS/MS) [27]. As far as we know, this type of sample, i.e., hydrogels of CMC in combination with disaccharides, were not tested using DART/TOF-MS; thus, we examined this possibility. Consequently, in this study, the influence of disaccharides (trehalose and sucrose) addition on the polyphenol composition of chokeberry hydrogels was investigated, and the possibilities of using direct analysis in real-time ionization with time-of-flight mass spectrometry (DART/TOF-MS) methods and HPLC-DAD method for the detection of polyphenols in prepared samples were evaluated.

## 2. Results and Discussion

### 2.1. Optimization of the DART-TOF/MS Method and DART-TOF/MS Analysis

The first part of the DART-TOF/MS analysis was the optimization of the ionization temperature. It is important to select the proper temperature as the DART ion source can operate at temperatures between 50 °C and 500 °C, and low temperatures are often not sufficient for the ionization of target compounds, while high temperatures can cause the degradation of thermolabile compounds [28]. Auto sampler velocity, placement of rods, flow of the carrier gas, and other DART ion source settings (needle, grid, fragmentor, and skimmer potential) were set according to previously published works [29,30]. To select the optimal ionization temperature, the intensity of one of the samples (2% CMC) was measured within a temperature range from 200 to 400 °C, with a temperature difference of 50 °C.

**Figure 1:**
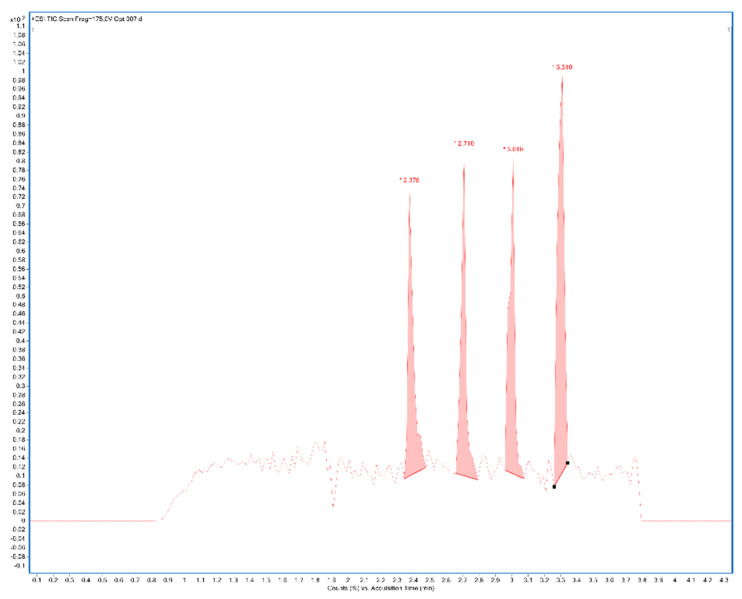
Example of the chronogram obtained after measuring in four parallels (TIC—total ion current chromatogram) of sample 2% CMC after manual integration in positive ionization mode (CMC: carboxymethylcellulose).

**Figure 2:**
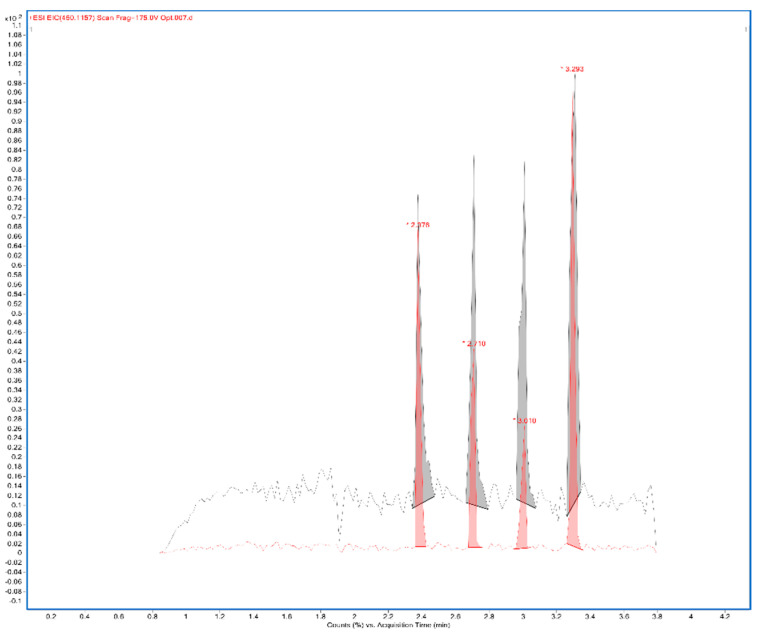
Extracted mass 450.1157 from the chronogram of 2% CMC sample (CMC: carboxymethylcellulose).

**Figure 3:**
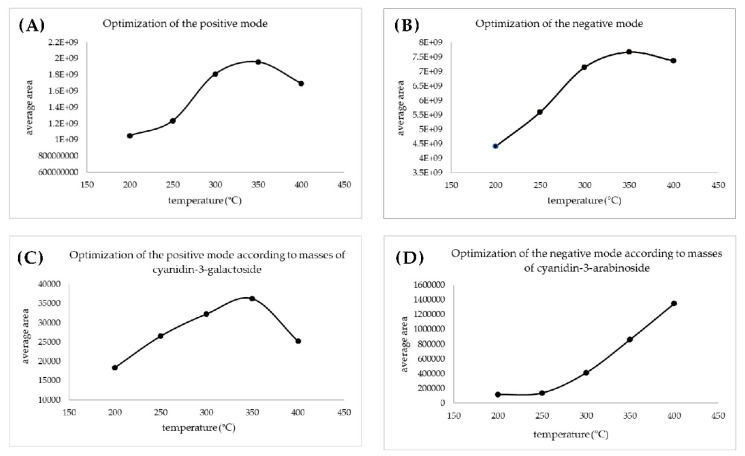
Optimization of the ionization temperature for positive (**A**) and for the negative mode (**B**) based on the areas obtained from the integration of the chronogram; optimization of the ionization temperature based on the areas obtained from the integration of extracted masses for positive (**C**) and for negative (**D**) mode.

A total ion current chronogram was obtained for each sample (Figure 1). Each sample was measured twice in four parallels, and thus, eight parallel measurements were obtained for each sample. For each sampling rod, a record was seen on the chronogram and after the extraction of the specific mass peaks were shown (Figure 2). The mass spectra of the sample in positive and negative mode were extracted. Each peak was then integrated, areas were obtained, and the point in the graph represents the average from eight measurements (Figure 3A,B). In the positive mode, a specific mass for cyanidin-3-galactoside with an *m*/*z* value of 450.1157 [M+H] ^+^ was extracted from the chronogram. Optimal signal-to-noise response was observed for an ionization temperature of 350 °C (Figure 3C). In the negative mode, a specific mass for cyanidin-3-arabinoside with an *m*/*z* value of 453.0594 [M-H]^-^ was extracted, and the optimal signal-to-noise response was observed at the temperature of 400 °C as the most data could be detected in the maximum (Figure 3D).

**Figure 4:**
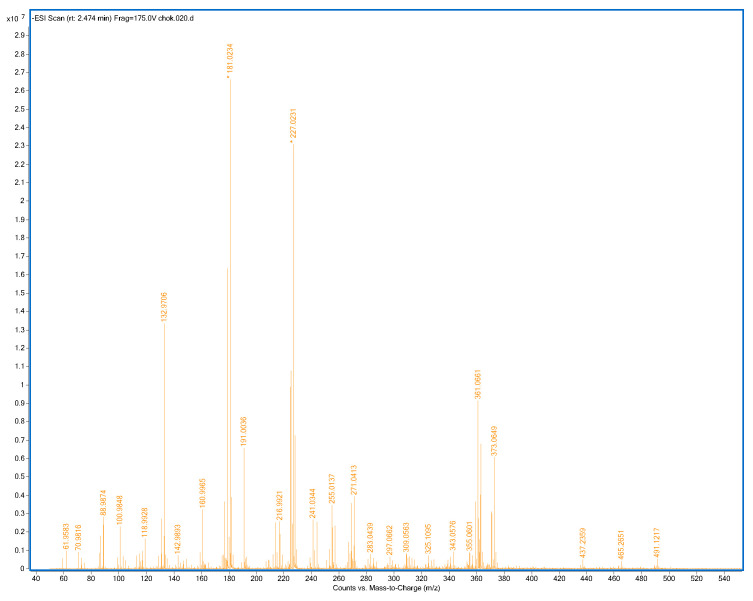
Mass spectrum of 2% CMC sample, negative mode, and ionization temperature 400 °C (CMC: carboxymethylcellulose).

All samples and standards were measured in positive and negative ion modes at optimized temperatures. Table 1 shows the measured ion, summary formula, calculated mass, experimental mass (*m*/*z*), and mass error (ppm) of standards of polyphenols evaluated by DART-TOF/MS in positive mode, while Table 2 shows this data for negative mode. The DART-TOF/MS spectrum of the one sample (2% CMC) in negative mode is presented in Figure 4.

**Figure 5:**
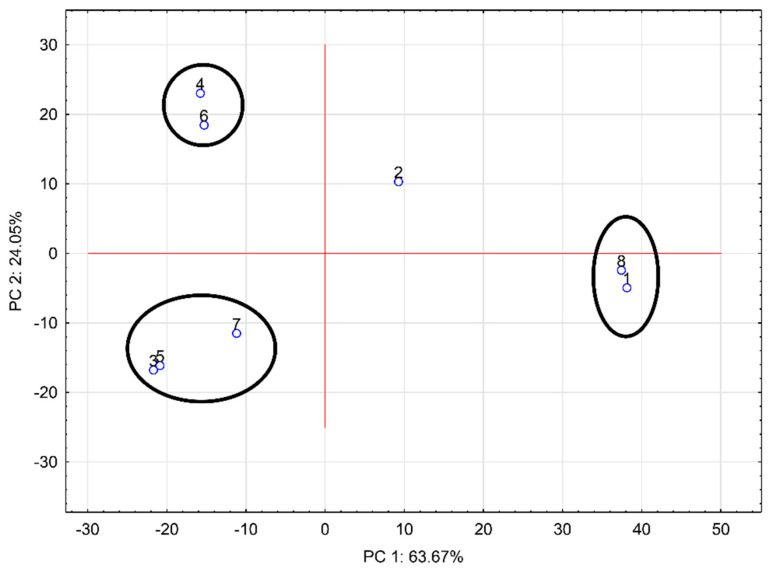
Principal component analysis of the mass spectra of all samples; negative mode, ionization temperature 400 °C (1: 2% CMC; 2: 2% CMC-30% S; 3: 2% CMC-30% T; 4: 2% CMC-40% S; 5: 2% CMC-40% T; 6: 2% CMC-50% S; 7: 2% CMC-50% T; 8: chokeberry juice; CMC: carboxymethylcellulose; S: sucrose; T: trehalose).

The measured mass spectra were processed using principal component analysis (PCA). The 2D PCA plot based on the mass spectra of prepared hydrogels and chokeberry juice obtained in the negative ion mode is shown in Figure 5. As sample 2% CMC (1) is the formulated cluster with chokeberry juice (8), it can be concluded there was not a significant difference in the composition of these two samples. Hydrogels with the addition of trehalose (3: 2% CMC-30% T, 5: 2% CMC-40% T, and 7: 2% CMC-50% T) were gathered within a relatively small cluster. Samples with the addition of sucrose (2% CMC-40% S (4) and 2% CMC-50% S (6)) were in a compact group, while 2% CMC-30% S (2) was separated. PCA factors were 63.67% and 24.05%, which means the PCA showed a significant difference between the individual samples. From PCA it is evident there is a significant difference in the composition of the samples without disaccharide addition and those with sucrose or trehalose addition. It can be concluded the addition of disaccharides altered the composition of chokeberry hydrogels. These results also confirm the potential of using DART-TOF/MS to perform rapid screening of prepared hydrogels. For atypical samples, further chemical analysis can be applied [28].

In the present study, DART-TOF/MS analysis was used for fingerprinting as this method is mainly perceived as a qualitative technique. This is due to the high fluctuation of absolute signal intensities within repeated measurements [31]. This method enables obtaining the mass spectrum of the analyzed sample without or with minimum pretreatment and in real time [28]. Furthermore, samples are analyzed at atmospheric pressure and in the open laboratory environment, which makes this method useful for the analysis of the ingredients of the plant material [32]. Phenolic compounds were also evaluated using this technique in the study by Chernetsova et al. [33]. Precise quantification by this method cannot be achieved using external calibration. The only way is by adding the internal standard in the form of stable isotope-labeled analyte analogues to the sample directly as they can eliminate the fluctuation problem and compensate for matrix effects that occur in ambient ionization techniques. These labeled compounds are not available for a wide range of analytes, and those that are commercially available are often expensive and increase the cost of the analysis [31].

### 2.2. Polyphenols and Antioxidant Activity of Chokeberry Juice

Chokeberry juice used for the preparation of hydrogels was analyzed for total polyphenols, monomeric anthocyanins, concentration of individual polyphenols, and antioxidant activity determined using ABTS, DPPH, CUPRAC, and FRAP assays, and results are given in Table 3. Results obtained were in accordance with previously published studies [2,5].

### 2.3. Evaluation of Polyphenols in Hydrogels

In the current study, the influence of the disaccharide addition on total polyphenols, monomeric anthocyanins, individual polyphenols, and antioxidant activity of CMC hydrogels was investigated. Total polyphenols and monomeric anthocyanins determined spectrophotometrically are presented in Table 4. Comparison of results of hydrogels with chokeberry juice showed that degradation of polyphenols and anthocyanins occured due to thermal treatment of mixture during hydrogels preparation and interactions of components between each other. It was observed total polyphenols of prepared samples ranged from 22.92 g/kg (2% CMC-50% T) to 35.44 g/kg (2% CMC). The highest concentration was observed for the sample without disaccharide addition; thus, it can be concluded the addition of sucrose and trehalose negatively affected the concentration of total polyphenols in hydrogels. The same negative effect was observed for monomeric anthocyanins as the hydrogel without disaccharide addition (2% CMC) had the highest concentration of anthocyanins (27.77 mg/kg). The lowest concentration of monomeric anthocyanins was observed for 2% CMC-50% T, and it was 10.21 mg/kg. Similar results were obtained in the study of Kirakosyan et al. [34] where it was observed cherry products processed with sugars contained lower concentrations of total polyphenols and monomeric anthocyanins than those without sugar. Additionally, in the study of Kopjar et al. [17], orange jelly without addition of trehalose had a higher concentration of total polyphenols after preparation than jelly with trehalose addition. By comparing the effect of sucrose and trehalose on total polyphenols, it was observed in the case of 30% and 40% disaccharide addition, trehalose hydrogels showed higher concentrations of total polyphenols than sucrose hydrogels (31.81 g/kg for 2% CMC-30% T and 27.81 g/kg for 2% CMC-30% S; 27.46 g/kg for 2% CMC-40% T; and 25.16 g/kg for 2% CMC-40% S), except for the addition of 50% disaccharide when hydrogels with sucrose addition had higher concentrations of total polyphenols. The same positive effect of trehalose compared to other disaccharides was observed in the study of Vukoja et al. [18] where the addition of trehalose to blackberry cream fillings caused the highest concentration of total polyphenols in samples compared to sucrose and maltose. It was reported even though sucrose and trehalose are chemical isomers they act differently in complex matrices. There are several ways in which disaccharides affect stability of other components present in the system. Firstly, trehalose has a higher affinity for water than sucrose and binds water molecules, which causes higher changes in water structure and the creation of more homogeneous water solutions. Secondly, disaccharides cause steric hindrance, which enables a decrease of nucleophilic attacks of water on unstable components. Thirdly, trehalose is more stable regarding hydrolysis in comparison with sucrose, and unsaturated compounds can form stable intramolecular complexes with trehalose. It was concluded that conditions during processing affected interactions between components present in the fruit matrix; thus, retention or loss of polyphenols occurred [18,35,36,37,38,39,40].

To evaluate the concentrations of individual polyphenols in the samples, HPLC-DAD analysis was conducted, and obtained results are presented in Table 5. Five different polyphenols were identified and quantified. The most abundant polyphenol in all hydrogels was cyanidin-3-galactoside followed by the other anthocyanin present in the samples, cyanidin-3-arabinoside. Concentrations of cyanidin-3-galactoside ranged from 53.67 mg/kg to 282.13 mg/kg for 2% CMC-50% T and 2% CMC, respectively. The same trend was observed for the concentrations of cyanidin-3-arabinoside, and 2% CMC had the highest concentration of this anthocyanin (62.18 mg/kg), while 2% CMC-50% T hydrogel had the lowest concentration (10.55 mg/kg). The higher binding of cyanidin-3-galactoside could be associated with the number of present hydroxyl groups in the anthocyanin structure [40], as cyanidin-3-galactoside contains one hydroxyl group more than cyanidin-3-arabinoside in its structure. By comparing only samples with sucrose and trehalose addition, it can be observed only in the case of hydrogels with 30% disaccharide, trehalose addition had a more positive effect than sucrose on concentrations of cyanidin-3-galactoside and cyanidin-3-arabinoside (241 mg/kg for 2% CMC-30% T and 212.75 for 2% CMC-30% S). From the results, it is evident the degradation of anthocyanins occurred during preparation of hydrogels due to increased temperature applied during the preparation of the samples and interactions that occurred between CMC and sucrose or trehalose. There are two mechanisms by which thermal degradation of anthocyanins occurs: hydrolysis of the 3-glycoside linkage and hydrolytic opening of the pyrilium ring. The first mechanism leads to the formation of more unstable aglycon, while the second one leads to the formation of substituted chalcone and its degradation to brown compounds with polyphenolic nature [41]. Concentrations of rutin ranged from 0.96 mg/kg for 2% CMC-50% T to 3.75 mg/kg for 2% CMC. The addition of disaccharides caused a decrease of rutin concentration. For neochlorogenic acid, the concentration range was from 4.26 mg/kg to 10.64 mg/kg for 2% CMC-50% T and 2% CMC, respectively. Hydrogel 2% CMC also contained the highest concentration of chlorogenic acid (11.75 mg/kg), while for other samples, statistical difference for concentration of chlorogenic acid was not observed. The results of the previously published study showed phenolic acids from purple carrot juice bind to cellulose but not to an equal extent [42]. Different polyphenols bind to cellulose and its derivates with different rates and extents but have similar binding behavior. The interactions between components from the cell wall, which is namely composed of cellulose, hemicelluloses, and pectic polysaccharides, depend on the chemical characteristics of polyphenols and the physical properties of the polysaccharides [43]. The combination of hydrogen bonds and hydrophobic interactions are the most common types of bonds between cellulose and individual polyphenols. Additionally, molecules with higher molecular weight bind to cellulose to a greater extent [40]. Polyphenols that have more aromatic rings in their structure bind more strongly to cellulose than polyphenols with only one aromatic ring in their structure [43]. This may explain why higher concentrations of anthocyanins compared to other groups of polyphenols were preserved in CMC hydrogels. Additionally, the hydrophilic character of CMC and disaccharides and the presence of water in the system had influence on the maximum binding capacity of polyphenols. Results showed in the presence of disaccharides, lower amounts of polyphenols were evaluated that could be explained with interactions between CMC molecules and additionally between CMC and disaccharides, leading to the decreases of interactions between CMC and polyphenols.

### 2.4. Evaluation of Antioxidant Activity of Hydrogels

There are various methods for the determination of antioxidant activity, but as different methods are based on different mechanisms of action, comparison of one method to another is not possible [44]. Antioxidant activity is never measured directly but by the effects of the antioxidant to control the degree of oxidation. Some methods involve different oxidation steps followed by the measurement of the response, which depends on the method used to evaluate the antioxidant activity [45]. In this study, four different assays were applied for the determination of antioxidant activity, and results obtained are presented in Table 6. The ABTS method is used to evaluate the reactivity of antioxidant samples in the presence of peroxides [45]. Antioxidant activity determined by ABTS ranged from around 21.00 μmol/100 g to 38.31 μmol/100 g. The highest value of antioxidant activity determined by this method was observed for the sample 2% CMC. The highest concentration of total polyphenols was also observed for this sample. From these results, it can be concluded the addition of disaccharides caused the decrease of antioxidant activity of the hydrogels. The antioxidant activity determined using the DPPH method showed the same trend as the results from the ABTS method. Values obtained ranged from around 22.00 μmol/100 g to 29.24 μmol/100 g. This method is simple and is widely used in various fields of chemistry to evaluate hydrophilic and lipophilic antioxidants [45]. The CUPRAC method is based on measuring the antioxidant activity correlated with the reduction of cupric (Cu^2+^) to cuprous (Cu^+^) ions [44]. Results obtained using the CUPRAC method ranged from 137.23 μmol/100 g (2% CMC-50% T) to 231.46 μmol/100 g (2% CMC). Results obtained by the FRAP method revealed the highest antioxidant activity had hydrogel without the addition of disaccharides (2% CMC); its antioxidant activity was 3.52 μmol/100 g. The FRAP method is commonly used to measure total antioxidant activity. However, it was reported FRAP values can vary significantly [45]. The addition of sucrose and trehalose also caused the decrease of antioxidant activity determined by the ABTS and FRAP methods in freeze-dried sour cherry puree investigated by Lončarić et al. [19]. The addition of these disaccharides also caused the decrease of antioxidant activity (DPPH and ABTS assays) of apple fiber/blackberry microparticles in the previously published study by Kopjar et al. [20]. Changes in antioxidant activity during food processing are possibly caused by interactions with other food components while they are mixed during food preparation [16]. It is known the measurement of antioxidant activity is essential to ensure quality of the functional foods and to evaluate the efficiency of food antioxidants in preventing diseases related to oxidative stress [44].

Correlation coefficients for the dependence of antioxidant activity determined using ABTS, DPPH, CUPRAC, and FRAP assays and the sum of total phenolic compounds, total anthocyanins, and total phenolic acids determined by HPLC-DAD were calculated and presented in Table 7. The highest correlation (0.8965) was observed for the ABTS assay, and the sum of total phenolic acids and the lowest correlation were determined for the CUPRAC assay and the sum of total anthocyanins (0.8191). In the study of Zhang et al. [46], it was reported high antioxidant activities are not only related to the content of total phenolic compounds but also to the content of total phenolic acids. Close correlations between antioxidant activity and total phenolic compounds were also detected in the study of Lončarić et al. [19].

Comparing the results of antioxidant activity of hydrogels and chokeberry juice, there is no high difference as it was observed for polyphenols and anthocyanins contents. The elevated temperature that was used for the preparation of hydrogels can cause various reactions, such as oxidation, polymerization, and/or condensation reactions of polyphenols. The consequences of these reactions are loss of polyphenols and/or change of their structure as well as formation of different compounds. Antioxidant potential of polyphenols depends on their denotation of hydrogen; consequently, the higher number of hydroxyl groups leads to the higher possibility of free radical scavenging activity. The chemical structure and spatial conformation can cause modification of the reactivity of the molecules [47,48,49]. A strong tendency of some polyphenols for polymerization can cause important structural changes leading to change of antioxidant potential of newly formed polymers. On the other hand, newly formed polymers can exhibit a decrease in antioxidant potential. This effect can be a result of the steric hindrance that can reduce the availability of the hydroxyl groups when the degree of polymerization exceeds a critical value [47,50,51,52,53,54]. Other reactions on polyphenols, such as oxidation or degradation reactions, can occur, also leading to a change in the antioxidant potential of compounds. One should not neglect the complexity of the matrix, i.e., the presence of other compounds and their structure. Other compounds present in the system cause interactions, which results in the modification of the structure and the availability of hydroxyl groups [55].

## 3. Materials and Methods

### 3.1. Chemicals

CMC was purchased from CP Kelco (Äänekoski, Finland). Sucrose was a product of Gram-mol (Zagreb, Croatia). Trehalose was obtained from Hayashibara doo (Nagase group, Tokyo, Japan). Hydrochloric acid was from Carlo Erba Reagents (Sabadell, Spain). Sodium carbonate was from T.T.T. (Sveta Nedelja, Croatia). Folin–Ciocalteu reagent and potassium persulfate were from Kemika (Zagreb, Croatia). Trolox, 2,2-diphenyl-1-picrylhydrazil, 2,2′-azino-bis(3-ethylbenzothiazoline-6-sulfonic acid) diammonium salt, and most of the standards used (rutin, quercetin, quercetin-3-D-glucoside, chlorogenic acid) were products of Sigma-Aldrich (St. Louis, MO, USA), while the standards of cyanidin-3-glucoside, cyanidin-3-galactoside, cyanidin-3-arabinoside, neochlorogenic acid, and quercetin-3-D-galactoside were products of Extrasynthese (Genay, France). Cupric chloride, neocuproine, and 2,4,6-tri(2-pyridyl)-s-triazine (TPTZ) were purchased from Acros Organic (Geel, Belgium). Methanol (HPLC grade) was from J.T. Baker (Deventer, Netherlands) and orthophosphoric acid (HPLC grade, >85%) was from Fisher Scientific (Longhborough, UK).

### 3.2. Preparation of Samples

Chokeberry juice was prepared by pressing the fruits and filtering the obtained mass. Hydrogels were prepared by adding 30%, 40%, and 50% of sucrose or trehalose to chokeberry juice. The juice was preheated on a magnetic stirrer to 60 °C. Afterwards, the addition of 2% of CMC caused the formation of hydrogels, which were complexed on a magnetic stirrer for 30 min. In the next step, the hot samples were poured into heated glass jars. After cooling, the samples were analyzed.

### 3.3. Extraction of Polyphenols

To perform extraction of polyphenols, 2 g of the sample and 20 mL of acidified methanol (methanol:hydrochloric acid ratio was 99:1) were mixed and homogenized. This mixture was left at room temperature for 24 h [56]. Extracts were filtered using PTFE 0.20 µm microfilters (Macherey-Nagel, Düren, Germany), and filtrates were used for spectrophotometric analyses and HPLC-DAD analysis.

To prepare samples for DART/TOF-MS analysis, 0.5 g of the sample was weighed, and 25 mL of methanol acidified with formic acid (1% formic acid in methanol) was added. To homogenize the samples, Ultra-Turrax (IKA T18 Basic; IKA Werke GmbH & Co., Staufen, Germany) was used. Homogenized extracts were filtered using PTFE 0.45 µm microfilters (OlimPeak, Teknokroma, Spain) into vials. The sampling DIP-it rods were dipped into the extracts and then placed to the auto sampler.

### 3.4. Direct Analysis in Real-Time Ionization with Time-Of-Flight Mass Spectrometry (DART/TOF-MS) Analysis

The DART unit includes the ionization source and the control electronics. In the measurement mode, helium gas (SIAD, Prague, Czech Republic; purity 5.5) was used, while in the standby mode, purified nitrogen gas using Agilent RMSN-4 universal trap and nitrogen generator (Peak Scientific, NM32 LA at 6.0 bar operating pressure) was used. The parameters of the DART ion source were set as follows: 3000 V needle potential, 350 V grid potential, 175 V fragmentor potential, 65 V skimmer potential, and 1 mm/s auto sampler velocity. These parameters were chosen according to a previously published study [57], and the temperature used was set by optimization. For sample feed, a 12 DIP-it auto sampler (IonSense, Saugus, MA, USA) with DIP-it sampling rods (IonSense, Saugus, MA, USA) was used. An API-TOF Reference Mass Solution Kit (Agilent Technologies, Santa Clara, USA) was used for tuning the TOF/MS. All sample measurement operations were controlled by Agilent Technologies B.04.00 MassHunter Acquisition Workstation software, and data were processed using Agilent Qualitative B.04.00 MassHunter Workstation Software. To obtain data for mass spectral studies, the total ion current chronogram was recorded in the range of 50–1000 *m*/*z*. Standards of polyphenols were prepared in the concentration range from 0.001 µg/mL to 100 µg/mL. All samples and standards (cyanidin-3-arabinoside, cyanidin-3-galactoside, cyanidin-3-glucoside, rutin, quercetin-3-D-galactoside, quercetin-3-D-glucoside, quercetin, neochlorogenic acid, chlorogenic acid) were analyzed four times in two parallels. They were measured in both positive and negative modes at ionization temperatures of 200–400 °C in increments of 50 °C.

### 3.5. Determination of Total Polyphenols, Monomeric Anthocyanins, and Antioxidant Activity

#### 3.5.1. Total Polyphenols and Monomeric Anthocyanins

The method by Singleton and Rossi [58] was slightly modified for spectrophotometric determination of total polyphenols. Briefly, 1.8 mL of deionized water was added to 0.2 mL of extract followed by the addition of 10 mL of Folin–Ciocalteu reagent (1:10) and 8 mL of sodium carbonate (7.5%). These mixtures were kept in the dark for 120 min and measured at 765 nm using a spectrophotometer (Cary 60, UV-VIS, Agilent Technologies, Santa Clara, CA, USA). Each sample was analyzed in triplicate. The obtained results were interpolated on a calibration curve created for gallic acid, and results were expressed as g of gallic acid equivalents per kg of hydrogel (g GAE/kg).

For determination of monomeric anthocyanins, the pH differential method was used. This method was previously described by Giusti et al. [59]. Firstly, 2.8 mL of 0.025 M KCl at pH 1 or 2.8 mL of 0.4 M sodium acetate at pH 4.5 were added to 0.2 mL of extract. All samples were analyzed in triplicate. Prepared mixtures were kept in the dark for 15 min, and absorbance was calculated using the following equation:

(1) $$A=\;{\left(A_{515}\;{-\;A}_{700}\right)}_{\mathrm{pH}\;1}\;-\;{\left(A_{515}\;{-\;A}_{700}\right)}_{\mathrm{pH}\;4.5}$$

where A_515_ denotes the absorbance read at 515 nm, and A_700_ is absorbance read at 700 nm. The concentration of monomeric anthocyanins was then calculated using formula:

(2) $$\mathrm{monomeric}\;\mathrm{anthocyanins}=\left({A\;\times \;M}_{W}\;\times \;\mathrm{DF}\;\times \;1000\right)/\left(\varepsilon \;\times \;l\right)$$

where Mw represents the molecular weight of cyanidin-3-glucoside (449.2 g/mol), DF is the dilution factor, ε is the molar absorptivity (26,900 L/mol cm), and l is the length of the cuvette (1 cm). The concentration of monomeric anthocyanins was calculated using data for cyanidin-3-glucoside and expressed as mg of cyanidin-3-glucoside per gram of hydrogel (mg cyanidin-3-glucoside/g).

#### 3.5.2. Antioxidant Activity (ABTS, DPPH, CUPRAC, FRAP Assays)

Antioxidant activity was evaluated using four different assays. For ABTS assay, 0.1 mL of extract was mixed with 3 mL of ABTS reagent (7 mM). The prepared mixture was kept in the dark, and absorbance was measured at 734 nm after 95 min. This method was previously described by Arnao et al. [60]. The DPPH method by Brand-Williams et al. [61] was slightly modified. Briefly, 0.2 mL of sample was mixed with 3 mL of DPPH solution (0.5 mM). The prepared mixture was kept in the dark and measured at 517 nm after 15 min. For determination of copper (II) reducing antioxidant activity, the CUPRAC assay was performed according to the method previously described by Apak et al. [62]. To conduct the analysis, 1 mL of CuCl_2_ (10 mM), neocuproine (7.5 mM) and ammonium acetate buffer (1 M, pH 7.0) were added to a glass tube, and then, the sample and distilled water were added to a total volume of 1.1 mL. The mixture was left for 30 min in the dark, and then, the absorbance was read at 450 nm. Determination of ferric reducing ability (FRAP) was conducted according to the Benzie and Strain method [63]. Shortly, 0.2 mL of extract was mixed with 3 mL of FRAP reagent. After the samples were kept in the dark for 30 min, absorbance was read at 593 nm. For all four assays, samples were analyzed in triplicate. The calibration curves were created for Trolox, and results were expressed as µmol of Trolox equivalents per 100 g of hydrogel (µmol TE/100 g).

### 3.6. High-Performance Liquid Chromatography-Diode Array Detection (HPLC-DAD) Method

For the evaluation of individual polyphenols in extracts and chokeberry juice, Agilent HPLC system 1260 Infinity II (Agilent technology, Santa Clara, CA, USA) was used. The system was equipped with a quaternary pump, a diode array detector (DAD), a vial sampler, and a Poroshell 120 EC C-18 column (4.6 × 100 mm, 2.7 µm). Orthophosphoric acid (0.1%) was used as mobile phase A, while methanol (100%) as used as mobile phase B. Injection volume was set to 5 µL, and flow rate was 1 mL/min. The following gradients were used: 0 min 5% B, 3 min 30% B, 15 min 35% B, 22 min 37% B, 30 min 41% B, 32 min 45% B, 40 min 49% B, 45 min 80% B, 48 min 80% B, 50 min 5% B, and 53 min 5% B. The method used was previously described by Buljeta et al. [64]. Before injection into the system, 1 mL of extract was filtered using a 0.20 µm PTFE syringe filter. Each sample was injected two times. Identification was done by comparing the retention times and UV-Vis spectra of peaks in the extracts. To confirm the identification, extracts were spiked with standards. The calibration curves were created for cyanidin-3-glucoside and cyanidin-3-arabinoside in the range from 5 to 300 mg/L, for chlorogenic acid from 25 to 500 mg/L, for neochlorogenic acid from 1 to 300 mg/L, for quercetin from 5 to 150 mg/L, and for rutin from 0.25 to 500 mg/L. The linearity of the curves was confirmed by R^2^ = 0.9969 for cyanidin-3-galactoside, R^2^ = 1 for cyanidin-3-arabinoside, R^2^ = 0.9993 for rutin; R^2^ = 0.9986 for neochlorogenic acid, and R^2^ = 0.999 for chlorogenic acid. The concentrations of individual polyphenols were expressed as mg of polyphenols per kg of hydrogel or chokeberry juice (mg/kg).

### 3.7. Statistical Analysis

All results were expressed as the mean values ± standard deviation. Statistical analysis was performed using software STATISTICA 13.1 (StatSoft Inc., Tulsa, OK, USA). Analysis of the variance (ANOVA) and Fisher’s least significant difference (LSD) with the significance defined at *p* < 0.05 were used for data analysis.

For grouping the data obtained by DART-TOF/MS analysis according to the *m/z* for a given abundance threshold, a macro was created in Excel 2016 (Microsoft). A detailed description of the work in the macro is described elsewhere [65]. The created table was ranked according to *m/z* descending, and thus, the assembled matrix was used for the principal component analysis (PCA) using Statistica 13.1 (StatSoft, Inc.). For statistical processing using PCA, 1000 of the most significant masses were taken in both positive and negative modes. The threshold (minimum abundance) for masses in positive mode was 5000 and in negative mode was 70,000.

## 4. Conclusions

Chokeberry hydrogels were prepared from CMC to investigate the influence of the disaccharide addition on total polyphenols, monomeric anthocyanins, individual polyphenols, and antioxidant activity. Results showed the addition of sucrose and trehalose negatively affected those parameters. Optimal measuring conditions of DART-TOF/MS analysis to measure polyphenols in chokeberry hydrogels determined in this study were in the negative mode at an ionization temperature of 400 °C. This method could be used for fingerprinting polyphenols. However, for the quantification of the polyphenols, the HPLC-DAD method is recommended.

To achieve higher retention of polyphenols in products, it is important to formulate the food systems properly, i.e., hydrogels from chokeberry juice and 2% CMC should be prepared without disaccharide addition. Prepared hydrogels could be used in the bakery and confectionery industry to improve the nutritional value of the products. Future studies should focus on the formulations of the final products and their overall quality.

## Figures and Tables

**Table 1:** Calculated and experimental masses for evaluated standards of polyphenols obtained by DART-TOF/MS in positive mode.

Polyphenol	Ion	Formula	Calculated Mass	Experimental Mass	Mass Error (ppm)
Cyanidin-3-arabinoside	[M+H]^+^	C_20_H_19_O_10_^+^	420.1051	420.1298	58.9
Cyanidin-3-galactoside	[M+H]^+^	C_21_H_21_O_11_^+^	450.1157	450.1406	55.3
Cyanidin-3-glucoside	[M+H]^+^	C_21_H_21_O_11_^+^	450.1157	450.1514	79.3
Rutin	[M+H]^+^	C_27_H_30_O_16_	611.1607	611.1737	21.3
Quercetin-3-D-galactoside	[M+H]^+^	C_21_H_20_O_12_	465.1028	465.1077	10.5
Quercetin-3-D-glucoside	[M+H]^+^	C_21_H_19_O_12_	464.0949	464.1400	97.2
Quercetin	[M+H]^+^	C_15_H_10_O_7_	303.0499	303.0519	6.6
Chlorogenic acid	[M+H]^+^	C_16_H_18_O_9_	355.1024	355.1021	−0.8
Neochlorogenic acid	[M+H]^+^	C_16_H_18_O_9_	355.1024	355.1073	13.8

**Table 2:** Calculated and experimental masses for evaluated standards of polyphenols obtained by DART-TOF/MS in negative mode.

Polyphenol	Ion	Formula	Calculated Mass	Experimental Mass	Mass Error (ppm)
Cyanidin-3-arabinoside	[M-H]^−^	C_20_H_19_O_10_^+^	418.0905	418.0074	−198.8
Cyanidin-3-galactoside	[M-H]^−^	C_21_H_21_O_11_^+^	448.1011	448.1221	46.9
Cyanidin-3-glucoside	[M-H]^−^	C_21_H_21_O_11_^+^	448.1011	448.0237	−172.8
Rutin	[M-H]^−^	C_27_H_30_O_16_	609.1461	609.0600	−141.4
Quercetin-3-D-galactoside	[M-H]^−^	C_21_H_20_O_12_	463.0882	463.0108	−167.2
Quercetin-3-D-glucoside	[M-H]^−^	C_21_H_19_O_12_	462.0804	462.0009	−172.1
Quercetin	[M-H]^−^	C_15_H_10_O_7_	301.0354	300.9780	−190.7
Chlorogenic acid	[M-H]^−^	C_16_H_18_O_9_	353.0878	353.0210	−189.2
Neochlorogenic acid	[M-H]^−^	C_16_H_18_O_9_	353.0878	353.0250	−177.9

**Table 3:** Evaluation of chokeberry juice polyphenols and antioxidant activity.

Total polyphenols (g GAE/L)	133.56 ± 0.06
Monomeric anthocyanins (mg cy-3-glu/L)	156.30 ± 0.79
Concentrations of individual polyphenols (mg/L)	
Cyanidin-3-galactoside	77.40 ± 0.54
Cyanidin-3-arabinoside	15.25 ± 0.11
Rutin	46.79 ± 0.59
Neochlorogenic acid	454.74 ± 0.00
Chlorogenic acid	381.65 ± 2.59
Antioxidant Activity (µmol TE/100 mL)	
ABTS	49.88 ± 0.54
DPPH	31.65 ± 0.01
CUPRAC	237.08 ± 2.03
FRAP	3.57 ± 0.01

GAE: gallic acid equivalents; cy-3-glu: cyanidin-3-glucoside; TE: Trolox equivalents; ABTS: (2,2-azino-bis(3-ethylbenzothiazoline-6-sulfonic acid)-radical scavenging activity; DPPH: (2,2-diphenyl-1-picrylhydrazyl)-free radical scavenging activity; CUPRAC: cupric reducing antioxidant capacity; FRAP: ferric reducing antioxidant power.

**Table 4:** Total polyphenols and monomeric anthocyanins of chokeberry hydrogels and chokeberry juice after preparation.

Sample	Total Polyphenols (g GAE/kg)	Monomeric Anthocyanins (mg cy-3-glu/kg)
2% CMC	35.44 ± 0.79 ^e^	27.77 ± 0.02 ^g^
2% CMC-30% S	27.81 ± 0.29 ^c^	23.73 ± 0.53 ^e^
2% CMC-30% T	31.81 ± 0.53 ^d^	26.52 ± 0.17 ^f^
2% CMC-40% S	25.16 ± 0.13 ^b^	21.91 ± 0.08 ^d^
2% CMC-40% T	27.46 ± 0.92 ^c^	17.29 ± 0.49 ^c^
2% CMC-50% S	27.40 ± 0.32 ^c^	13.87 ± 0.42 ^b^
2% CMC-50% T	22.92 ± 0.59 ^a^	10.21 ± 0.23 ^a^

CMC: carboxymethylcellulose; S: sucrose; T: trehalose; GAE: gallic acid equivalents; cy-3-glu: cyanidin-3-glucoside. Within the column, means followed by superscript different letters are significantly different at *p* ≤ 0.05 (ANOVA, Fisher’s LD).

**Table 5:** Concentrations of individual polyphenols in chokeberry hydrogels and chokeberry juice (mg/kg) determined using HPLC analysis.

Sample	Cy-3-gal	Cy-3-arab	Rut	NcA	ChA
2% CMC	282.13 ± 1.75 ^g^	62.18 ± 1.85 ^g^	3.75 ± 0.06 ^e^	10.64 ± 0.07 ^f^	11.75 ± 0.00 ^b^
2% CMC-30% S	212.75 ± 0.13 ^e^	47.99 ± 0.22 ^e^	2.27 ± 0.01 ^c^	7.33 ± 0.24 ^d^	9.22 ± 0.14 ^a^
2% CMC-30% T	241.00 ± 1.22 ^f^	53.93 ± 0.44 ^f^	2.83 ± 0.04 ^d^	6.38 ± 0.07 ^e^	8.87 ± 0.06 ^a^
2% CMC-40% S	187.34 ± 1.00 ^d^	42.58 ± 0.15 ^d^	1.63 ± 0.07 ^b^	4.93 ± 0.07 ^c^	7.66 ± 0.02 ^a^
2% CMC-40% T	168.19 ± 1.05 ^c^	38.00 ± 0.20 ^c^	1.77 ± 0.04 ^b,c^	4.96 ± 0.03 ^c^	7.78 ± 0.01 ^a^
2% CMC-50% S	59.19 ± 0.11 ^b^	12.29 ± 0.20 ^b^	1.35 ± 0.08 ^a,b^	5.51 ± 0.07 ^b^	7.38 ± 0.03 ^a^
2% CMC-50% T	53.67 ± 0.19 ^a^	10.55 ± 0.07 ^a^	0.96 ± 0.03 ^a^	4.26 ± 0.01 ^a^	7.36 ± 0.01 ^a^

CMC: carboxymethylcellulose; S: sucrose; T: trehalose; Cy-3-gal: cyanidin-3-galactoside; Cy-3-arab: cyanidin-3-arabinoside; Rut: rutin; NcA: nechlorogenic acid; ChA: chlorogenic acid. Within the column, means followed by superscript different letters are significantly different at *p* ≤ 0.05 (ANOVA, Fisher’s LD).

**Table 6:** Antioxidant activity (FRAP, CUPRAC, DPPH, and ABTS assays) of chokeberry hydrogels and chokeberry juice.

Sample	ABTS (µmol TE/100 g)	DPPH (µmol TE/100 g)	CUPRAC (µmol TE/100 g)	FRAP (µmol TE/100 g)
2% CMC	38.31 ± 0.38 ^d^	29.24 ± 0.50 ^d^	231.46 ± 0.96 ^g^	3.52 ± 0.03 ^f^
2% CMC-30% S	26.69 ± 0.58 ^b^	24.69 ± 0.28 ^b^	168.18 ± 1.61 ^d^	2.55 ± 0.02 ^c^
2% CMC-30% T	31.59 ± 0.85 ^c^	27.15 ± 0.65 ^c^	202.58 ± 1.76 ^f^	3.07 ± 0.01 ^e^
2% CMC-40% S	21.74 ± 0.34 ^a^	22.28 ± 0.62 ^a^	152.45 ± 0.56 ^b^	2.26 ± 0.02 ^b^
2% CMC-40% T	27.22 ± 0.39 ^b^	24.99 ± 0.63 ^b^	170.49 ± 1.11 ^e^	2.62 ± 0.03 ^d^
2% CMC-50% S	21.15 ± 0.10 ^a^	22.84 ± 0.89 ^a^	158.61 ± 1.11 ^c^	2.19 ± 0.02 ^a^
2% CMC-50% T	20.98 ± 0.48 ^a^	22.04 ± 0.50 ^a^	137.23 ± 1.21 ^a^	2.16 ± 0.01 ^a^

CMC: carboxymethylcellulose; S: sucrose; T: trehalose; TE: Trolox equivalents; ABTS: (2,2-azino-bis(3-ethylbenzothiazoline-6-sulfonic acid)-radical scavenging activity; DPPH: (2,2-diphenyl-1-picrylhydrazyl)-free radical scavenging activity; CUPRAC: cupric reducing antioxidant capacity; FRAP: ferric reducing antioxidant power. Within the column, means followed by superscript different letters are significantly different at *p* ≤ 0.05 (ANOVA, Fisher’s LD).

**Table 7:** Calculated correlation coefficients of the dependence of antioxidant activity determined using ABTS, DPPH, CUPRAC, and FRAP assays and the sum of total phenolic compounds, total anthocyanins, and total phenolic acids determined by HPLC-DAD.

r^2^	ABTS	DPPH	CUPRAC	FRAP
Total phenolic compounds	0.8538	0.8315	0.8296	0.8540
Total anthocyanins	0.8439	0.8217	0.8191	0.8446
Total phenolic acids	0.8965	0.8706	0.8868	0.8787

## Data Availability

Data is contained within the article.
